# Phytohormone ethylene-responsive *Arabidopsis* organ growth under light is in the fine regulation of Photosystem II deficiency-inducible *AKIN10* expression

**DOI:** 10.1038/s41598-017-02897-5

**Published:** 2017-06-05

**Authors:** Geun-Don Kim, Young-Hee Cho, Sang-Dong Yoo

**Affiliations:** Department of Life Sciences, Division of Life Sciences, KOREA University, Seoul, Korea

## Abstract

For photoautotrophic plants, light-dependent photosynthesis plays an important role in organismal growth and development. Under light, *Arabidopsis* hypocotyl growth is promoted by the phytohormone ethylene. Despite well-characterized ethylene signaling pathways, the functions of light in the hormone-inducible growth response still remain elusive. Our cell-based functional and plant-system-based genetic analyses with biophysical and chemical tools showed that a chemical blockade of photosystem (PS) II activity affects ethylene-induced hypocotyl response under light. Interestingly, ethylene responsiveness modulates PSII activity in retrospect. The lack of ethylene responsiveness-inducible PSII inefficiency correlates with the induction of *AKIN10* expression. Consistently, overexpression of *AKIN10* in transgenic plants suppresses ethylene-inducible hypocotyl growth promotion under illumination as in other ethylene-insensitive mutants. Our findings provide information on how ethylene responsiveness-dependent photosynthetic activity controls evolutionarily conserved energy sensor AKIN10 that fine-tunes EIN3-mediated ethylene signaling responses in organ growth under light.

## Introduction

A photosynthetic organism plant converts solar energy into organic molecules and self-supplies cellular energy under light. Intuitively, plants largely determine their cell fates in response to light information. For example, the plant hormone ethylene-inducible organ growth is differentially regulated by light conditions^1^. More specifically, *Arabidopsis* hypocotyl growth is inhibited and promoted in response to ethylene in the absence and presence of light, respectively^2–5^. The light-dependent ethylene-inducible hypocotyl growth promotion results mainly from cellular enlargement rather than proliferation; hence cell division cycle regulation may be excluded in their controlling mechanisms^2^.

Ethylene is perceived by ETHYLENE RESPONSE1 (ETR1) and four other closely related gene products that act as the hormone receptor in *Arabidopsis* ^6–8^. The high-molecular-weight ethylene receptor complexes consist of the hormone receptors, CONSTITUTIVE TRIPLE RESPONSE1 (CTR1), and other unknown component(s). In the absence of ethylene, CTR1 directly interacts with and phosphorylates another membrane protein ETHYLENE INSENSITIVE2 (EIN2)^9–11^. In the presence of ethylene, the inverse agonistic receptor complexes^12^ block the CTR1 function on EIN2, enabling an unknown protease to cleave the C-terminal half of nonphosphorylated EIN2 (EIN2C). EIN2C moves into the nucleus, stabilizes key transcription factors, EIN3 and EIN3-LIKE1 (EIL1), and triggers ethylene-responsive gene expression^8, 10^.

For ethylene-inducible *Arabidopsis* hypocotyl growth in light, EIN3 specifically binds to the PHYTOCHROME-INTERACTING FACTOR3 (PIF3) promoter and induces the gene expression^5^. Two other homologs of PIF3 seem to play key roles in modulation of the organ growth as well^13^. Despite well-characterized ethylene-dependent regulatory pathways, how light information is involved in the hormone-inducible growth promotion remains largely elusive. For instance, how light-labile PIF3 and its homologs can contribute to the light-dependent organ growth is still puzzling.

ETR1 is thought to have originated from the chloroplast genome, but its cellular functions have not been widely studied in terms of plastid activity^14^. Recently, ethylene insensitivity has been implicated in decreasing photosynthetic outputs through the transcriptional down-regulation of the most abundant enzyme involved in the first commitment step of carbon fixation RUBISCO expression^15, 16^. Therefore, ethylene insensitivity, resulting in low photosynthetic activity, must lead to low energy status in plant cells along the light and dark cycle of the day.

Intracellular energy deprivation is known to activate the two isoforms of the evolutionarily conserved energy sensor Snf1-related protein kinase (SnRK1), namely, ARABIDOPSIS KINASE (AKIN)10 and AKIN11 in *Arabidopsis thaliana*, that can turn catabolic and anabolic pathways on and off, respectively, in order to sustain cellular viability^17^. The chemical blockade of the photosystem (PS) II pathway with 3-(3,4-dichlorophenyl)-1,1-dimethylurea (DCMU), as well as the intervention condition of mitochondrial aerobic respiration by hypoxia, potently induces energy depletion and readily activates AKIN10 activity in *Arabidopsis* ^18–20^. However, ethylene sensitivity has never been examined as a potential factor of cellular energy status by means of its regulation of chloroplast activity.

This gap in our knowledge results partly because further examination of ETR1 function using the dominant ethylene-insensitive *Arabidopsis etr1–1* has been hampered because it encloses an unexpected abnormal chloroplast structure, which may or may not interfere with photosynthetic activity. The rigorously studied *etr1–1* turned out to be harboring a second site mutation on *ACCUMULATION AND REPLICATION3* (*ARC3*)^21^, which is involved in the positioning of plastid division sites^22^. This chloroplast abnormality has, thus, disallowed the unambiguous understanding of the ETR1-dependent ethylene function in the modulation of chloroplast/photosynthetic activity, which results in cellular energy modulation.

Spectroscopic analysis of chlorophyll fluorescence emission is a well-established tool to measure the photochemical efficiency of PSII in chloroplasts of higher plants^23–26^. Pulsed amplitude-modulated (PAM) fluorimetry has been the technique of choice for such chlorophyll fluorescence analysis in leaves. This analysis provides information about the quantum yield of PSII, the relative size of the reduced quinone pool, and the extent of nonphotochemical quenching^27–31^. More recently, chlorophyll fluorescence lifetime (CFL) analysis has also been applied to measure the photosynthetic kinetics^32, 33^. Time-correlated chlorophyll fluorescence analysis of individual chloroplasts was monitored for *Arabidopsis* and *Alocasia* leaves under a two-photon excitation fluorescence lifetime imaging microscope (FLIM) with femtosecond pulses^34^. Such FLIM-based data correlated well with ensemble-based data in *Synechocystis* sp PCC 6803^35^. However, the FLIM-based CFL analysis has not been applied to live single cells of multicellular higher plants so far.

*Arabidopsis* leaf mesophyll protoplast is a versatile cellular system that is acquired after enzymatic removal of cell walls^36–38^. Leaf mesophyll protoplasts have robust physiological cellular responses to extracellular signals, and their genetic constituents are readily manipulated by transient expression of genes of interest. Microscopic images taken from mesophyll protoplasts also provide useful information about the organization and development of subcellular organelles.

Here, we showed that PSII activity is involved in regulation of ethylene-inducible *Arabidopsis* hypocotyl growth in light. Then, we took advantage of the protoplast system to reconstitute ethylene insensitivity in live single cells and demonstrated that the lack of ethylene responsiveness causes PSII inefficiency, leading to cellular energy deprivation, which activates *AKIN10* expression. Consistently, AKIN10 activation suppresses ethylene-inducible hypocotyl growth in light. Our findings revealed a functional link between ethylene responsiveness-dependent PSII efficiency and the PSII efficiency-dependent cellular energy sensor *AKIK10* expression for the control of ethylene-inducible hypocotyl growth promotion under illumination.

## Results and Discussion

### Ethylene promotes hypocotyl growth through PSII regulation in light

**Figure 1:**
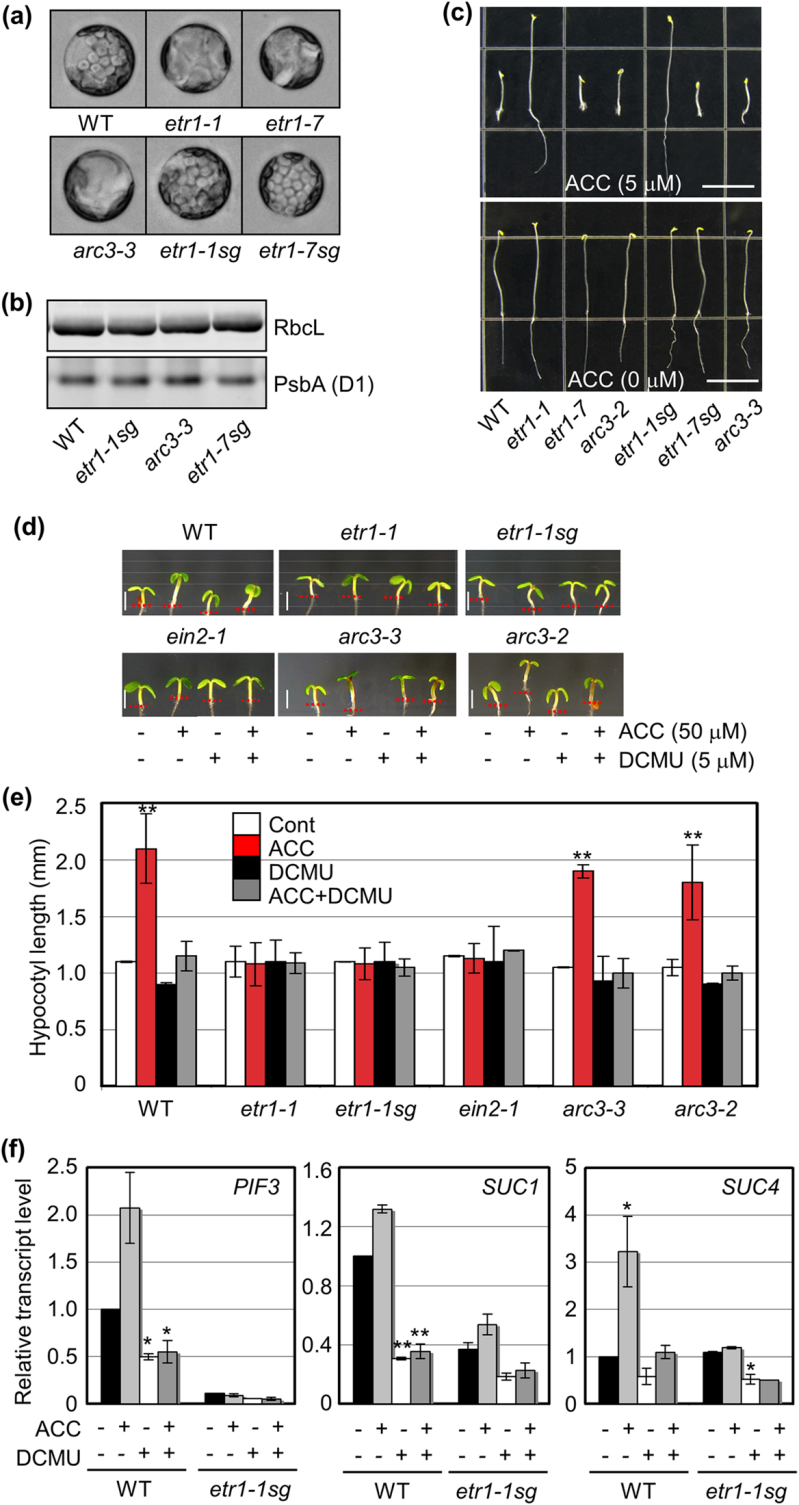
*etr1* and *arc3-3* alleles, respectively, control ethylene sensitivity and chloroplast morphology in the *etr1-1* background. (**a**) Chloroplast morphology in mesophyll protoplasts isolated from the leaves of WT, *etr1-1*, *etr1-7*, *arc3-3*, *etr1-1sg*, and *etr1-7sg*. *etr1-1* and *etr1-7* uncoupled from *arc3-3* are denoted as *etr1-1sg* and *etr1-7sg*, respectively. (**b**) Rubisco large subunit (RbcL) and PSII protein D1 (PsbA) protein levels are shown as representative photosynthetic enzymes. Protein blot analysis was performed with commercially available specific antibodies. (**c**) Triple response assay for WT, *etr1-1*, *etr1-7*, *arc3-2*, *etr1-1sg*, *etr1-7sg*, and *arc3-3*. Scale bar, 10 mm. (**d,e**) The light-controlled growth response of *Arabidopsis* hypocotyls observed (**d**) and measured (**e**) in the absence and presence of ACC with and without DCMU. Scale bar, 1 mm. *n* > 20. (**f**) Marker gene expression measured in WT and ethylene-insensitive *etr1-1sg* using qRT-PCR. All experiments conducted in triplicate with consistent results; the means of triplicate measurements are shown with standard error bars. ***p < 0.001, **p < 0.01, and *p < 0.05.

In our previous work an abnormal chloroplast structure was found in *etr1-1* resulting from a second site mutation on *arc3-*3^21^. Since *etr1-7* is an intragenic suppressor of *etr1-1* ^12^, *etr1-7* also enclosed abnormal chloroplasts in mesophyll cells similar to those of *etr1-1* (Fig. 1a). To separate the *etr1-1* and *etr1-7* alleles from the second mutation *arc3-3*, *etr1-1* and *etr1-7* were crossed with the wild type (WT; Col-0). Individual allele-specific mutants were isolated without *arc3-3* and named as *etr1-1sg* and *etr1-7sg*, respectively. Each genetic background was confirmed by using derived cleaved amplified polymorphic sequences (dCAPS) analysis and DNA sequencing (Fig. S1).

Unlike WT, several large chloroplasts with an abnormal structure were observed in protoplasts of ethylene insensitive *etr1-1* and ethylene hypersensitive *etr1-7* (Fig. 1a). Without the *arc3-3* allele, many small chloroplasts were observed in *etr1-1sg* and *etr1-7sg* as in WT, confirming that *arc3-3* caused the chloroplast abnormality. In WT, *etr1-1sg*, *etr1-7sg*, and *arc3-3*, the accumulation of two representative photosynthetic proteins, PSII D1 (PsbA) and Rubisco large subunit (RbcL) were detected at a similar level by protein blot analysis using specific antibodies (Fig. 1b). Thus, both the PSII protein complex and the downstream metabolic-output machinery in photosynthesis seem to be normal in *etr1-1sg* and *etr1-7sg*.

In the ethylene response, etiolated *Arabidopsis* WT seedlings grown on a full-strength Murashige and Skoog (MS) medium containing 1% (w/v) sucrose manifested a triple response of shortened hypocotyls and roots and exaggerated apical hook formation in the presence of an ethylene precursor 1-aminocyclopropane-1-carboxylic acid (ACC, Fig. 1c). The *etr1-1sg* exhibited ethylene insensitivity with its elongated hypocotyls in the same manner as *etr1-1*. Furthermore, *etr1-7sg* exhibited the same ethylene hypersensitive phenotype as *etr1-7*. All results indicated that the second site mutation on *ARC3* within *etr1-1* only affects the chloroplast phenotype, and the mutation on *ETR1* is critical for determining ethylene sensitivity in *etr1-1* and *etr1-7*.

In the light, *Arabidopsis* WT hypocotyl growth promotion was pronounced on an MS-deficient growth medium in the presence of 50 µM ACC^3, 4^, but not those of ethylene insensitive *etr1-1*, *etr1-1sg*, and another well-characterized strong ethylene insensitive mutant *ein2-1* (Fig. 1d,e). On the other hand, the hypocotyl growth promotion of two *arc3* alleles, *arc3-2* and *arc3-3*, was again pronounced in the presence of ACC and was similar to that of WT. To further investigate photosynthesis functions uncoupled from other effects of light on organ growth promotion, *Arabidopsis* hypocotyl growth was quantitatively analyzed in the presence and absence of DCMU, which blocks the PSII pathway^39^. Surprisingly, ethylene dependent hypocotyl growth promotion in light was largely suppressed in the presence of DCMU (Fig. 1d,e). Since DCMU is a sensitive blocker that interrupts the electron-transport chains in photosynthesis, its loss-of-photosynthetic activity may modulate cellular energy status and ethylene-dependent hypocotyl elongation under light.

To understand the molecular basis of light-grown *Arabidopsis* seedling responses to ethylene/ACC in terms of PSII regulation, the expression pattern of marker genes including skotomorphogenesis-related *PIF3* ^5^, photosynthate/sucrose-responsive *SUCROSE TRANSPORTER1* (*SUC1*) and *SUC4* ^40^ were examined using quantitative reverse transcriptase-dependent real-time PCR (RT-qPCR) with total RNA extracted from the shoots of 5-day-old WT and *etr1-1sg* seedlings grown in light. The expression of *PIF3*, *SUC1* and *SUC4* was induced by ACC in WT, but not in ethylene insensitive *etr1-1sg* (Fig. 1f). Moreover, the ACC-inducible marker gene expression was largely suppressed in WT by DCMU. Because EIN3-induced PIF3 plays main regulatory role in hypocotyl growth elongation, regulation of EIN3 level would result in the modulation of ACC/ethylene dependent hypocotyl growth promotion under light. All results suggest that the receptor-dependent ethylene signaling controls EIN3-dependent gene expression leading to hypocotyl growth promotion and perhaps PSII activity is involved in the ethylene-inducible organ growth regulation.

Sugars, the products of photosynthesis, function not only as energy resources, but also as signaling molecules that modulate the expression of genes such as *SUC1* and *SUC4* ^40^. To examine whether sugar signaling plays a role in ethylene-inducible hypocotyl growth promotion under light, hypocotyl growth of *gin2-1*, the plant glucose sensor *HEXOKINASE1*-null mutant, was monitored under light. The hypocotyls of *gin2* grew more or less like those of WT (L*er*) when grown in combinations of ACC and DCMU under light (Fig. S2); thus, glucose/sugar as a nutrient resource, but not as an intracellular signal that antagonizes ethylene signaling, apparently has a major role in connecting PSII activity in chloroplasts to ethylene-inducible hypocotyl growth promotion under light. In line with this notion, exogenous 1% (w/v) sucrose could pronounce ACC-dependent and -independent hypocotyl growth promotion in Col-0, but not in *ein2-1* and *ein3eil1* blocking EIN3-dependent ethylene signaling. This is consistent with our observation that EIN3-dependent PIF3 is necessary in *Arabidopsis* hypocotyl elongation in the presence of ACC/ethylene under light. Moreover PSII-dependent photosynthetic activity may modulate EIN3 and its downstream regulator PIF3 in ethylene-inducible hypocotyl elongation under light (Fig. S3).

### FLIM-based CFL measures PSII activity at single cell resolution

**Figure 2:**
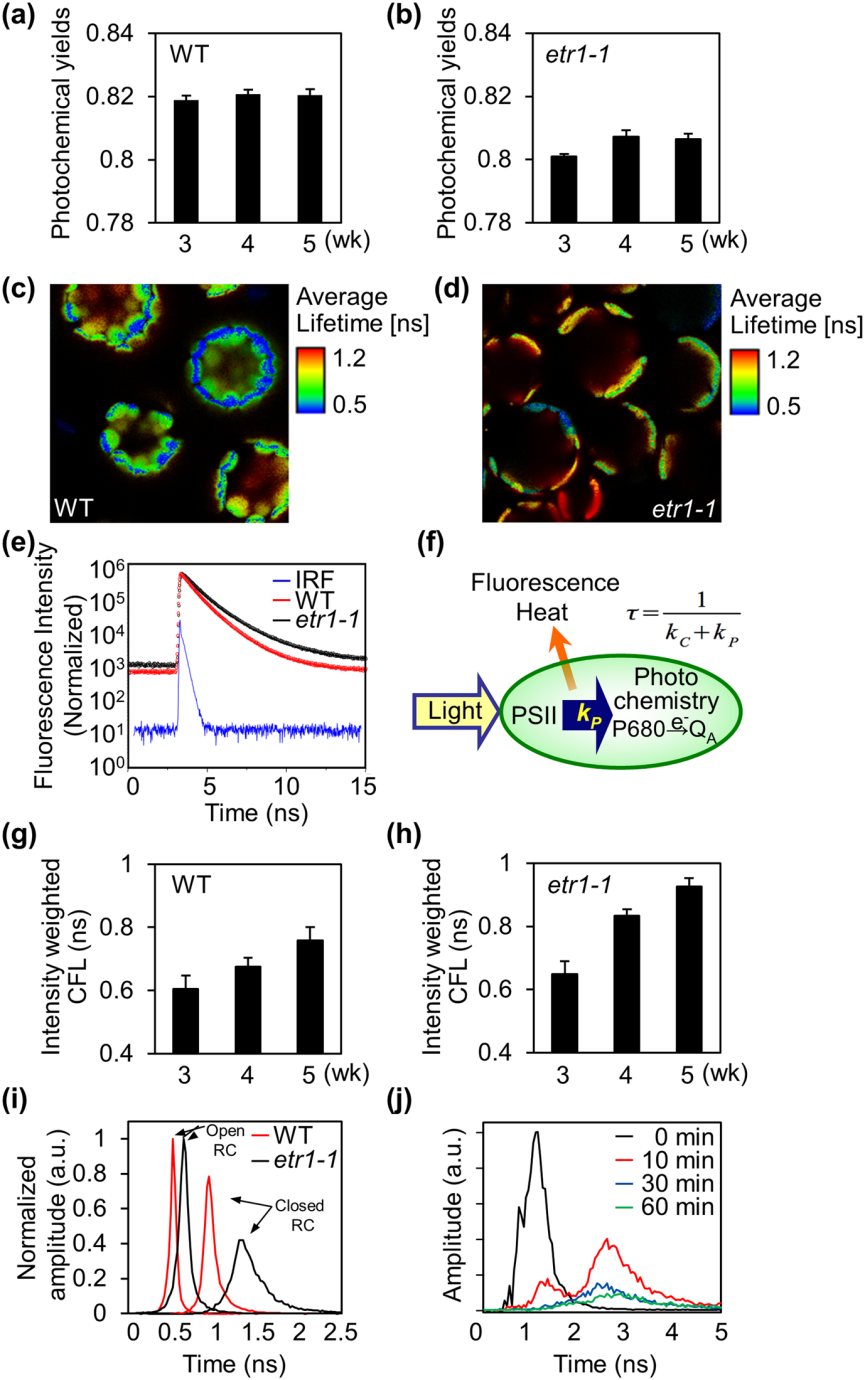
Differences in chlorophyll fluorescence emission between ethylene-sensitive WT and ethylene-insensitive *etr1-1* in *Arabidopsis*. (**a**,**b**) Photochemical yields (F_v_/F_m_) were measured using PAM fluorimetry in WT (**a**) and *etr1-1* (**b**) leaves 3 to 5 weeks after germination. (**c**,**d**) Chlorophyll fluorescence lifetimes (CFLs) -color-coded cellular images from leaf mesophyll protoplasts of WT (**c**) and *etr1-1* (**d**) were obtained using a time-correlated and space-resolved single photon-counting FLIM technique. Image sizes are 200 μm × 200 μm. (**e**) Chlorophyll fluorescence decay of *etr1-1* (black) and WT (red) are shown with instrumental response function (IRF, blue). CFL values were obtained using FLIM and then analyzed using the Symphotime Program. (**f**) Working model of photo-energy dissipation. (**g**,**h**) CFLs were measured using FLIM in WT (**g**) and *etr1-1* (**h**) mesophyll protoplasts isolated from leaves at 3 to 5 weeks after germination. (**i**) Normalized amplitude of CFL components from mesophyll protoplasts of WT and *etr1-1*. (**j**) Amplitudes of CFL components from WT protoplasts in the presence of DCMU.

Next we investigated whether ethylene responsiveness could affect PSII activity. First, we measured chlorophyll fluorescence using traditional pulse-amplitude modulation (PAM) fluorimetry and expressed these values as photochemical yields (F_v_/F_m_). Because each leaf of a plant ages differently, only the third and fourth leaves from 3- to 5-week-old soil-grown plants were used for analyses. F_v_/F_m_ was always higher in WT than in *etr1-1* at 3 to 5 weeks after germination (Fig. 2a,b).

We then measured PSII activity in the chloroplasts at single-cell resolution. To do so, an FLIM technique was applied to obtain CFLs in mesophyll protoplasts freshly isolated from mature leaves of ethylene sensitive WT and ethylene insensitive *etr1-1*. When color-coded CFLs were superimposed over leaf cell images, many small chloroplasts were largely shown to be bluish-green in WT protoplasts, but several large chloroplasts were yellowish-red in *etr1-1* protoplasts (Fig. 2c,d). These results indicated that chlorophyll fluorescence decays rather quickly in the chloroplasts of WT than in those of *etr1-1*. The convoluted intensity-weighted average CFLs of WT and *etr1-1* were estimated to be ca.797 ps and ca.1144 ps, respectively (Fig. 2e, Table 1). The CFL value obtained from WT protoplasts was much shorter than the values obtained from isolated antenna complexes, which were around 2 to 4 ns^41^. This may imply that photo energy transfer to the reaction center occurs rather quickly and efficiently in the cellular environment than in isolated light-harvesting complexes.

**Table 1:** Average lifetimes of chlorophyll fluorescence in *Arabidopsis* WT and *etr1-1*.

	A_1_^a^	τ_1_ (ns)	A_2_^a^	τ_2_ (ns)	τ_int.average_ (ns)^b^
WT	63.59	0.55	35.41	1.24	0.797
*etr1-1*	51.23	0.78	48.77	1.53	1.144

^a^ ${A}_{i}={C}_{i}{\tau }_{i}/\sum _{{\rm{i}}}{C}_{i}{\tau }_{i}$, where *C*_*i*_ is the amplitude at time zero. ^b^Intensity-weighted average lifetime, ${{\rm{\tau }}}_{{\rm{int}}{\rm{.avg}}}=\sum _{{\rm{i}}}{A}_{i}{\tau }_{i}$. *A biexponential decay function for deconvolution was used to calculate the amplitude of lifetime components and the average lifetime. Biexponential decay function ${\rm{I}}({\rm{t}})={C}_{1}{e}^{-\frac{t}{{\tau }_{1}}}+{C}_{2}{e}^{-\frac{t}{{\tau }_{2}}}$. Deconvolution function ${\rm{I}}({\rm{t}})={\int }_{-\infty }^{t}IRF(t^{\prime} )\{{C}_{1}{e}^{-\frac{t-{t}^{^{\prime} }}{{\tau }_{1}}}+{C}_{2}{e}^{-\frac{t-{t}^{^{\prime} }}{{\tau }_{2}}}\}dt^{\prime}$.

CFL reflects the efficiency of PSII activity in the experiment. PSII fluorescence is predominant at 700(+/−20) nm and nearly 100 times greater than that of PSI at room temperature. Furthermore, the plastoquinone pool is fully oxidized by dark incubation before CFL analysis^25^. Under such conditions, CFL values most likely echo the quenching efficiency of the PSII pathway by energy transfer to the reaction center, i.e., photochemical quenching^42, 43^. Therefore, when PSII efficiency is low, photochemical quenching is delayed/slow and CFL becomes longer in *etr1-1* than in WT (Fig. 2c–e). The differences in CFLs between WT and *etr1-1* were consistent throughout different developmental stages from 3 (mature) to 5 weeks (just before bolting), indicating that ethylene sensitivity/responsiveness modulates PSII activity throughout plant leaf development (Fig. 2g,h). In summary, both FLIM- and PAM fluorimetry-based chlorophyll fluorescence analyses consistently suggest that PSII photochemical activity in abnormal chloroplasts of ethylene-insensitive *etr1-1* plants is less efficient than in normal chloroplasts in ethylene-sensitive WT plants.

To further compare FLIM-based CFL and PAM fluorescence analysis, we tried to obtain photochemical yields with FLIM-based fluorescence analysis. When the chlorophyll fluorescence of PSII was measured in mesophyll protoplasts, parts of reaction centers must be closed by laser excitation. In the results, fluorescence values can be attributed to two different states of the reaction centers, open and closed. In WT and *etr1-1*, these two peaks were found under optimized fitting conditions (Fig. 2i). By referring to previous studies on the CFL of leaves and isolated chloroplasts, shorter (~500 ps) and longer (~1000 ps) peaks in WT were identified for reaction centers at open (τ_open_) and partially closed states, respectively (Fig. 2i)^44–47^. Interestingly, fluorescence values at both peaks were always relatively longer in *etr1–1* than in WT. When DCMU that chemically closes PSII pathway was applied, a new fluorescence component of 2700 ps was identified for reaction centers at completely closed state (τ_closed_) in WT (Fig. 2j). Then, photochemical yields was calculated as 0.81 as following F_v_/F_m_≈ (τ_open_^−1^ − τ_close_^−1^)/τ_open_^−1^ ^48^. This F_v_/F_m_ value calculated from CFL values was close to the values obtained by PAM fluorimetry (Fig. 2a), supporting its fidelity in analyzing PSII activity. The advancement of cellular imaging microscopy now allows specific observations of cellular structures and their functions *in vivo*. Its application to the measurement of chlorophyll fluorescence from chloroplasts in a single cell enables the accurate and dynamic detection of PSII photochemical efficiency at high resolution in live single cells of a higher plant, *Arabidopsis*.

### Ethylene responsiveness modulates the chlorophyll fluorescence lifetime in PSII

**Figure 3:**
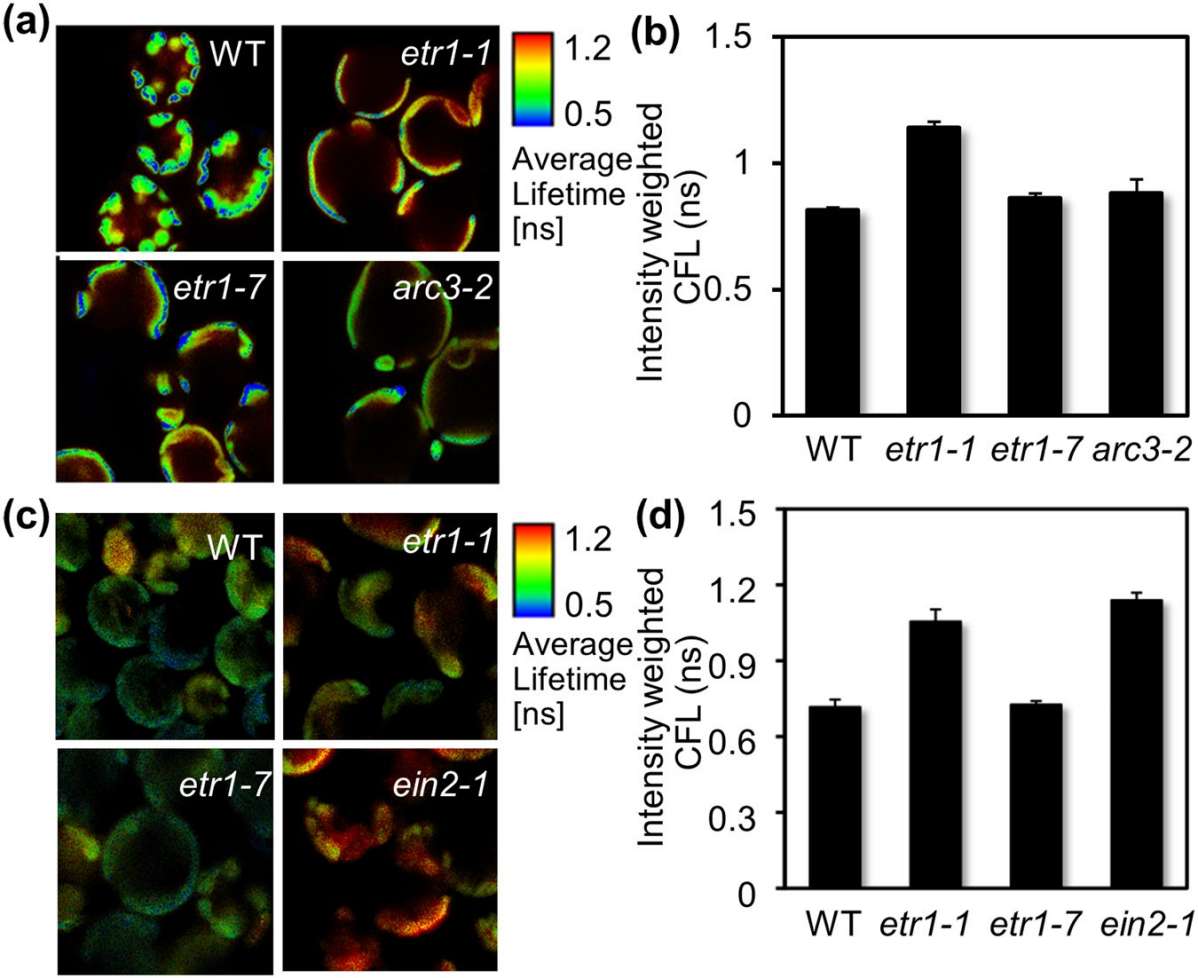
ETR1-dependent ethylene insensitivity modulates the CFL of PSII. (**a**,**b**) CFL-color-coded cellular images (**a**) and values (**b**) from leaf mesophyll protoplasts of WT, *etr1-1*, *etr1-7*, and *arc3-2* were obtained using a commercial FLIM device. (**c**,**d**) CFL-color-coded cellular images (**c**) and values (**d**) of leaf mesophyll protoplasts of WT, *etr1-1*, *etr1-7*, and *ein2-1* plants were obtained using an in-house fabricated FLIM device. All experiments were repeated three times with consistent results. The means of at least three measurements are shown with standard error bars.

Next we examined whether the chloroplast abnormality observed in *etr1-1* may affect the chlorophyll fluorescence of PSII. Unlike *etr1-1*, the CFLs of *etr1-7* and *arc3-2* were similar to those of WT (Fig. 3a,b). The *arc3-2* allele produces defective chloroplast division, resulting in development of giant chloroplasts as in *etr1*-*7* ^21, 49^. We then examined whether ethylene responsiveness affects the photochemical efficiency of PSII. A FLIM device was fabricated in house and used to monitor the CFLs of other strong ethylene insensitive *ein2-1* protoplasts, in addition to ethylene sensitive WT, ethylene insensitive *etr1*-*1*, and ethylene hypersensitive *etr1*-*7*. CFLs were again longer in *etr1*-*1* and *ein2*-*1* than in WT and *etr1*-*7* (Fig. 3c,d) indicating that the CFL of PSII is under the control of ethylene responsiveness.

**Figure 4:**
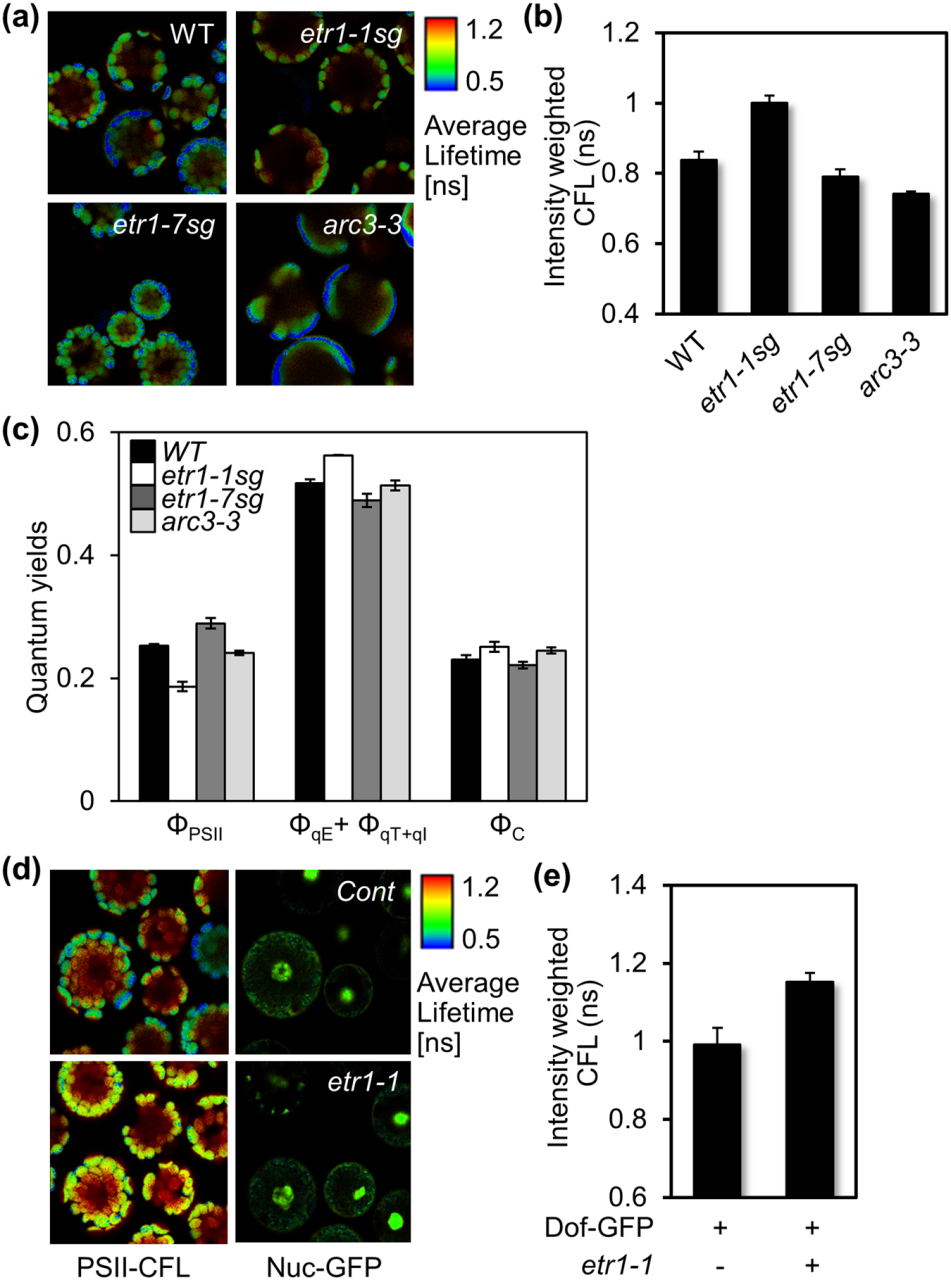
Ethylene responsiveness-coupled PSII activity modulates hypocotyl growth under light. (**a,b**) CFL-color-coded cellular images (**a**) and values (**b**) are shown for mesophyll protoplasts isolated from the leaves of WT, *etr1-1sg*, *etr1-7sg*, and *arc3-3* plants. (**c**) Chlorophyll fluorescence was measured in WT, *etr1-1sg*, *etr1-7sg*, and *arc3-3* specimens using PAM fluorimetry. (**d**,**e**) CFL-color-coded cellular images (**d**) and values (**e**) of WT leaf mesophyll protoplasts after reconstituting the lack of ethylene responsiveness. Protoplasts were cotransfected with either an effector *etr1-1* or the N-terminal half of YFP as an effector control and a nuclear GFP, and then incubated for 6 h. Protoplasts were then selected by nuclear GFP expression and subjected to FLIM analysis. All experiments were repeated three times with consistent results. The means of at least three measurements are shown with standard error bars.

To further verify our original observation that ethylene responsiveness modulates the chlorophyll fluorescence of PSII (Fig. 2), FLIM- and PAM fluorimetry-based analyses were conducted with mesophyll protoplasts isolated from *etr1-1sg* and *etr1-7sg*, and *arc3-3* (Fig. 4a). As shown in *etr1-1* (Fig. 3a,b), CFL was also longer in *etr1-1sg* than in WT, *etr1-7sg*, and *arc3-3* (Fig. 4a,b). Consistently, the quantum yields of PSII were relatively lower in *etr1-1sg*, but higher in *etr1-7sg*, than those in ethylene sensitive WT (Fig. 4c). The photochemical efficiency of *arc3-3* that is segregated from *etr1-1* was similar to that of the WT (Fig. 4a-c), confirming that abnormal chloroplast structure does not affect PSII activity. As in *etr1-1* (Fig. 3e), the quantum yields of NPQ in *etr1-1sg* were relatively higher than those in WT (Fig. 4c). Both FLIM and PAM fluorescence data reliably demonstrated that ethylene responsiveness, but not chloroplast structure, modulates the photochemical efficiency of PSII in the chloroplasts.

To substantiate that the lack of ethylene responsiveness leads to photochemical inefficiency of PSII, changes in CFL were measured from WT protoplasts concomitantly with *etr1-1* expression. Protoplasts were co-transfected with a construct encoding a nuclear marker-GFP that harbored DOF1a^50^, allowing for the selection of transfection-positive ones. CFL was relatively longer in WT protoplasts expressing *etr1-1* than in those expressing the N-terminal end of split-YFP as a control (Fig. 4d), indicating that the reconstitution of lack of ethylene responsiveness by *etr1-1* expression results in a longer CFL of PSII, i.e., low photochemical efficiency.

### Photochemical activity affects ethylene-inducible hypocotyl elongation under light through the cellular energy sensor SnRK1/AKIN10

**Figure 5:**
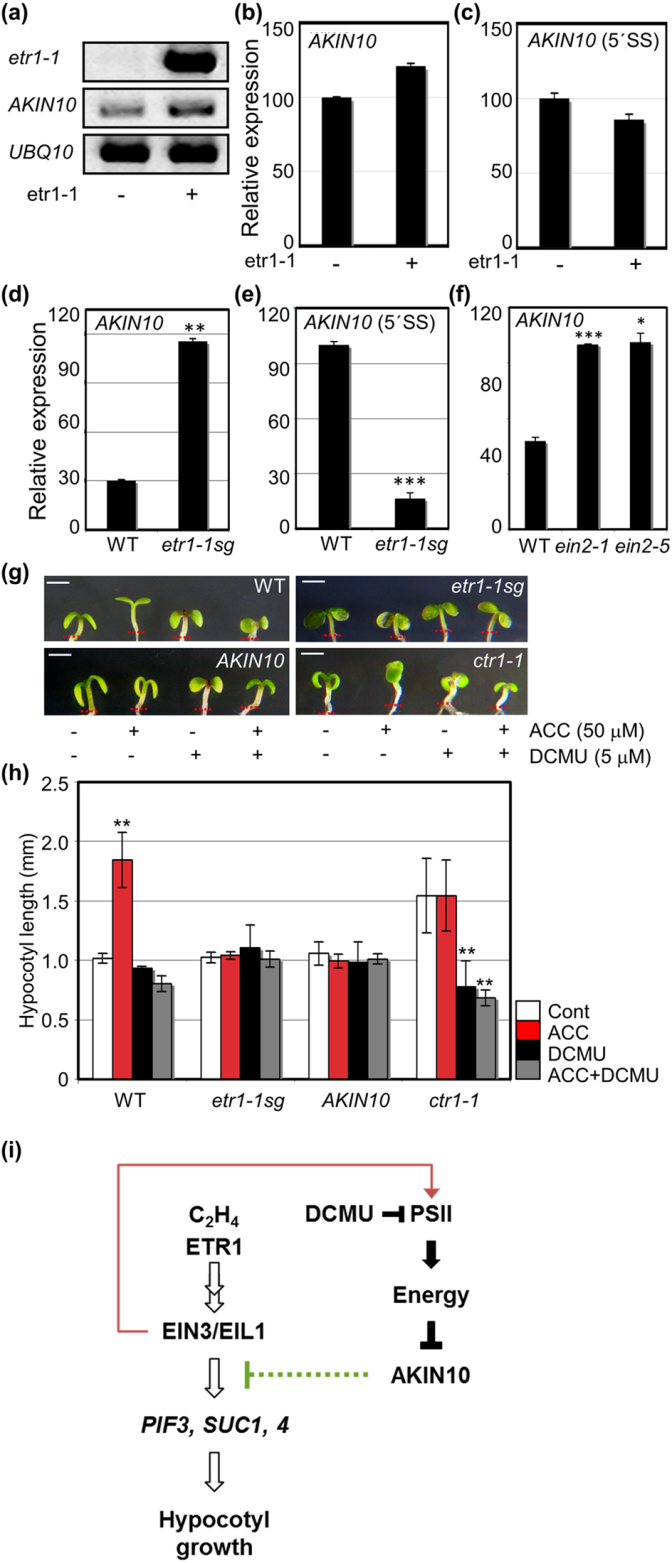
Lack of ethylene responsiveness induces *AKIN10* expression, which inhibits ethylene-inducible hypocotyl growth promotion under light. (**a**) *AKIN10* expression was monitored in mesophyll protoplasts transfected with and without *etr1-1* construct using semi-quantitative RT-PCR. *UBQ10* served as a control. Experiments were duplicated with consistent results. A representative result is shown. (**b**,**c**) Mature mRNA (**b**) and pre-mRNA (**c**) of *AKIN10* expression was monitored in mesophyll protoplasts transfected with and without *etr1-1* construct using RT-qPCR. (**d**,**e**) Mature mRNA (**d**) and pre-mRNA (**e**) of *AKIN10* expression was monitored in WT and *etr1-1sg* using RT-qPCR. (**f**) Mature mRNA of *AKIN10* expression was monitored in WT, *ein2-1* and *ein2-5* using RT-qPCR. (**g**,**h**) The light-controlled growth response of hypocotyls of WT, *etr1-1sg*, *AKIN10-*expressing transgenic seedlings, and *ctr1-1* were observed (**g**) and measured (**h**) in the absence and presence of ACC with and without DCMU. Scale bar, 1 mm. *n* > 20. All experiments conducted in triplicate with consistent results; the means of triplicate measurements are shown with standard error bars. ***p < 0.001, **p < 0.01, and *p < 0.05. (**i**) Working model of ethylene-inducible hypocotyl growth promotion under the fine control of ethylene responsiveness-dependent PSII activity in the light.

Since ethylene responsiveness controls PSII efficiency (Figs 2–4) and has a regulatory role in photosynthesis outputs^15, 16^, we reasoned that inefficient PSII activity caused by the lack of ethylene responsiveness might affect the cellular energy supply. To test this possibility, the cellular energy stress sensor *AKIN10* expression was monitored in WT protoplasts transfected with an *etr1-1* construct using a semi-quantitative RT-PCR. Notably, *AKIN10* expression was clearly increased by the lack of ethylene responsiveness imposed by *etr1-1* expression (Fig. 5a). More specifically mature *AKIN10* mRNA expression increased (Fig. 5b), but its pre-mRNA that retains the first intron decreased (Fig. 5c) in our RT-qPCR analysis.

To further examine the notion of ethylene responsiveness-dependent *AKIN10* expression, we monitored *AKIN10* expression in genetically predisposed ethylene insensitive *etr1-1sg* together with ethylene-sensitive WT. Mature *AKIN10* mRNA transcripts accumulated to a much higher level in *etr1-1sg* than in WT (Fig. 5d). Pre-mRNA transcripts of *AKIN10*, however, accumulated to a relatively lower level in *etr1-1sg* than in WT (Fig. 5e). These consistent but conditional results indicated that post-transcriptional regulation seemingly plays a role in *AKIN10* induction under the lack of ethylene responsiveness in a condition sensitized to their differential photosynthetic capacities. The mature *AKIN10* mRNA accumulated again more in both ethylene-insensitive *ein2-1* and *ein2-5* than in WT (Fig. 5f), supporting that ethylene responsiveness modulates *AKIN10* expression, perhaps through photosynthetic activity regulation. In line with this, mature mRNA of *AKIN10* accumulated highly in the presence of DCMU (Fig. S4a). Moreover, such induction of *AKIN10* expression seemingly leads to protein kinase activation, because the expression of AKIN10 target genes *DIN1* and *DIN6* was consistently induced in *etr1-1sg* (Fig. S4b) and also in WT only in the presence of DCMU (Fig. S4c). Taken together, the lack of ethylene responsiveness leads to PSII inefficiency, resulting in cellular energy deprivation to induce *AKIN10* expression and its kinase activation.

To genetically examine whether AKIN10 modulates ethylene-inducible hypocotyl growth under light, hypocotyl growth of transgenic plants expressed with *AKIN10* (ref. 19) was observed in the presence of ACC under light together with WT, ethylene-insensitive *etr1-1sg*, and ethylene-constitutive responsive *ctr1-1*. The WT hypocotyl growth was again promoted in the presence of ACC/ethylene under light, but not in *AKIN10*-expressing transgenic plants much as for *etr1-1sg* (Fig. 5g,h). Consistently, the organ growth was increased in *ctr1-1* in the absence and presence ACC under light^3, 4^, thereby reflecting the constitutive ethylene responsive nature of the genetic background. Interestingly, both ACC-dependent and independent growth promotion of *ctr1-1* was repressed by DCMU as in WT (Fig. 5g,h). Taken all together, these results strongly support that lack of ethylene responsiveness affects PSII activity and modulates hypocotyl growth promotion by regulating *AKIN10* expression under light conditions.

In this study we took advantage of a time-correlated and space-resolved fluorescence microscopic technique to characterize CFLs inversely reflecting PSII activity *in vivo* utilizing uniform and abundant mesophyll protoplasts isolated from mature *Arabidopsis* leaves. The CFL-based PSII activity in live single cells correlates well with chlorophyll fluorescence analysis using conventional PAM fluorimetry analysis at the tissue level. Here, we have described that *Arabidopsis* hypocotyl growth is under the regulation of ethylene signaling pathway (Fig. 5i). Ethylene responsiveness modulates PSII-dependent photosynthesis activity, controlling cellular energy sensor *AKIN10* expression. Cellular AKIN10 activity could involve in negative modulation of EIN3-mediated ethylene responsive gene expression. In turn ethylene responsiveness could secure its signaling responses by assisting full activation of PSII efficiency. Further studies on the functional mechanisms of ethylene signaling underlying the regulation of photosystem II efficiency is needed to yield novel strategies for more efficient solar-energy harvesting in higher plants.

## Materials and Methods

### Plant materials and genetic analysis

Plants were grown in soil at 24 °C for 20 to 22 days under 60 μmol/m^2^/s light intensity with a 12-h photoperiod. *Arabidopsis* Columbia-0 served as the wild-type (WT) and *etr1-1*, *etr1-7*, *arc3-2* (SALK_057144), *ein2-1*, and *ein2-5* mutant plants were used for experiments. The *arc3-3* allele was segregated from the *etr1-1* allele (*etr1-1sg*) or *etr1-7* allele (*etr1-7sg*). The genetic backgrounds of the segregated mutants were confirmed using dCAPS markers and DNA sequencing. Homozygous lines were identified by PCR with dCAPS primers (5′-TTTGGTGCTTTTATCGTTCCAT-3′ and 5′-TCCGGTTTCTTCCTGAGTTC-3′ for *etr1-1*, digested using *Nde*I; 5-TGGAGCAACTCATCTTATTAACTTCCG-3′ and 5′-TCCGGTTTCTTCCTGAGTTC-3′ for *etr1-7*, digested using *Hpa*II; and 5′-ATGGTTCCGAAATTCGCTTA-3′ and 5′-AAACTACCTCTTCTAGACGCTTCC-3′ for *arc3-3*, digested using *Hpa*II).

The ethylene response assay of etiolated seedlings was performed in complete darkness for 3 days with or without ACC. After 4 days of stratification, seeds were germinated on MS agar plates supplemented with 1% sucrose to ensure uniform germination.

For hypocotyl elongation assays, seedlings were grown on water-agar plates (without added salts or sugars) containing either no ACC or 50 µM ACC and/or 5 µM DCMU for 5 days under photon irradiance of 80 µmol·m^−2^ s^−1^. Experiments were repeated at least three times with 50 seedlings each time.

### Transient expression of Arabidopsis mesophyll protoplasts

Protoplast isolation and transient expression of genes of interest were carried out using previously described methods^22^. The *etr1-1* construct was generated by inserting cDNA between the *35SC4PPDK* promoter and the *NOS* terminator in a plant expression vector. The transfected protoplasts were incubated in W5 solution for 15 h and the CFL was measured.

### Photochemical efficiency measurement

MicroTime 200 (PicoQuant, Germany) was used for fluorescence lifetime imaging microscopy (FLIM) analysis. A diode-pulsed laser (470 nm, 40 MHz, 480 nW, LDH-P-C-470B/PDL 800-B; PicoQuant) was used to excite the chlorophyll, and emissions from 665 to 685 nm were collected in 40,000-pixel mode. Images with Chi-squared values of 0.70 to 1.30 were used in the analysis. Protoplasts were placed on an inverted microscope without any actinic light. The lifetime data of living protoplasts were collected according to the manufacturer’s recommendations. The collection time was approximately 1 min for each protoplast image. FLIM data were analyzed and fitted using the Symphotime Program provided by PicoQuant. The photochemical efficiency of PSII was measured using a portable plant efficiency analyzer (Hansatech Instruments, King’s Lynn, Norfolk, UK).

### PAM fluorimetry analysis

The quantum yield of PSII photochemistry, nonphotochemical quenching, and intrinsic decay were estimated using equations developed previously^4^. Chlorophyll fluorescence from bulk leaves was measured by FMS2 fluorimetry (Hansatech instruments, Norfolk, England). The saturating pulse and actinic light intensity were fixed at 5000 µE and 500 µE, respectively.

### RNA isolation and transcript accumulation analysis

ACC and/or DCMU-mediated gene regulation was investigated using monitoring marker gene expression in WT and *etr1-1sg* mutants. For gene expression analysis, total RNA was isolated using RNAiso Plus (Takara Bio, Otsu, Japan) from the shoots of 5-day-old seedlings grown on water-agar plates in the presence and absence of ACC and/or DCMU, and 1 µg of total RNA was used for cDNA synthesis using Moloney murine leukemia virus reverse transcriptase (Promega, Madison, WI, USA). Gene expression was measured using real-time PCR (CFX Connect^TM^ Real-Time System with C1000 Thermal Cycler, Bio-Rad), with seedlings grown on water-agar plates serving as controls. RT-qPCR was carried out using an iQ SYBR Green dye-supplemented PCR mix (Bio-Rad). Either *Tubulin4 (TUB4*, At1g04820) or *Elongation initiation factor4a* (*EIF4a*, At3g13920) transcripts were used as real-time PCR controls with gene-specific primers. Primer sequences are presented in Supplemental Table S1. The production of a single gene product by each primer set in the PCR reactions was validated before each experiment. Experiments were repeated three times (using more than 20 seedlings each time), with consistent results.

### Protein extraction and protein blot analysis

Total proteins were extracted from the third and fourth leaves of each plant using an extraction buffer containing 150 mM NaCl, 50 mM Tris-HCl (pH7.5), 5 mM EDTA, 1 mM DTT, 1% (v/v) Triton X-100, and complete protease inhibitor. Protein blot analysis was performed using 40 μg of total protein and protein amounts were quantified using the Bradford method (Bio-Rad). Anti-RbcL-rabbit or anti-PsbA-rabbit antibody (Agrisera) was used for detection.

## Electronic supplementary material

Supplementary information
