# Supplementary material for: Phytohormone ethylene-responsive Arabidopsis organ growth under light is in the fine regulation of Photosystem II deficiency-inducible AKIN10 expression
Source: Sci Rep. 2017 Jun 5;7:2767. doi: 10.1038/s41598-017-02897-5 (PMC5459816; doi:10.1038/s41598-017-02897-5)

**Phytohormone ethylene-responsive *Arabidopsis* organ growth under light is in the fine regulation of Photosystem II deficiency-inducible *AKIN10* expression**

Geun-Don Kim^1^, Young-Hee Cho^1^, Sang-Dong Yoo^*^

Department of Life Sciences, Division of Life Sciences, KOREA University, Seoul, Korea

***Corresponding Author:**

Sang-Dong Yoo

Department of Life Sciences

Division of Life Sciences

KOREA University

145 Anamro, Seongbuk-gu

Seoul, Korea, 02841

Tel: +82-2-3290-3401

Fax: +82-2-927-9028

E-mail: sangdong@korea.ac.kr

**Footnote: ^1^**Theses authors contribute equally to this study.

**
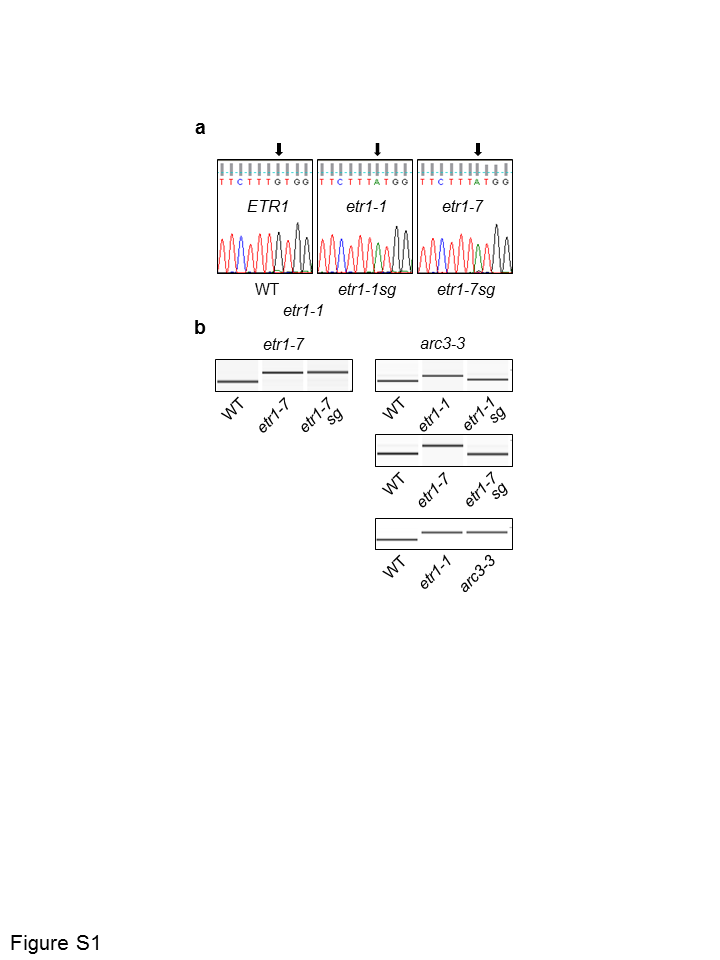
**

**Fig. S1. The segregation of *arc3-3* from *etr1-1* or *etr1-7.*** (**a**) DNA sequence analysis of WT, *etr1-1*, and *etr1-1sg.* (**b**) dCAPS marker analysis of *arc3-3* and *etr1* alleles.

**
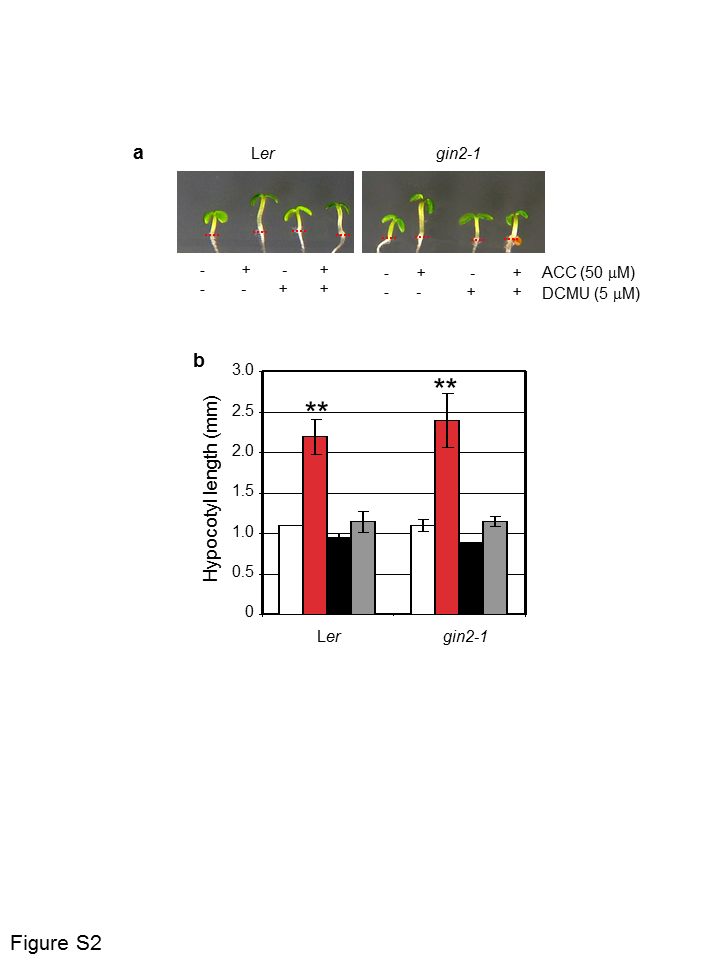
**

**Fig. S2. The light-controlled growth response of the glucose sensor HXK1-null mutant *gin2-1.* (a,b)** Light responses of *gin2-1* hypocotyls were observed (**a**) and measured (**b**) in the absence or presence of ACC with or without DCMU. L*er* was used as the WT plant. Experiments were repeated three times with consistent results. The means of triplicate experiments (*n* = 20) are shown with standard-error bars.

**
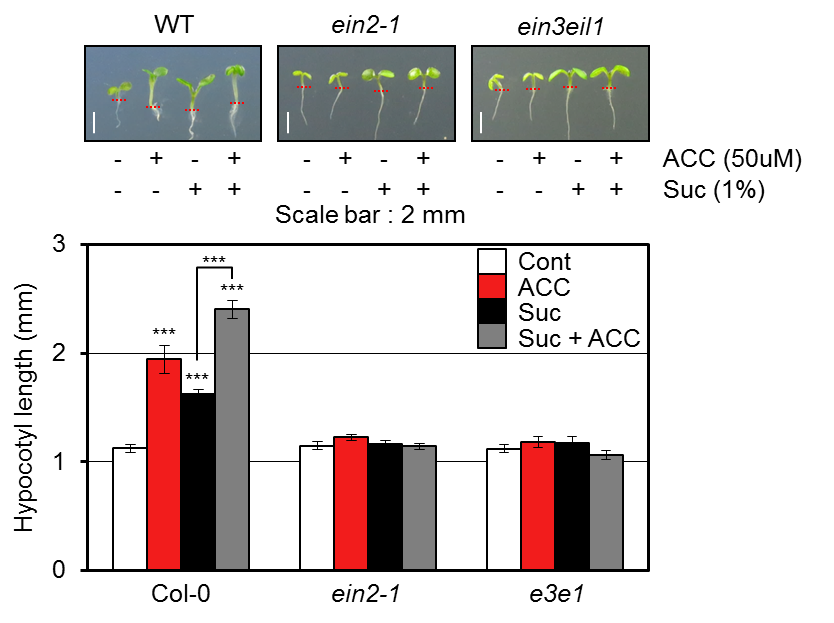
**

**Fig. S3. The sucrose-dependent hypocotyl growth response (a,b)** Growth responses of Col-0 and *ein2-1* hypocotyls were observed (**a**) and measured (**b**) in combination of ACC with and sucrose in half-strength MS agar media. Col-0 was used as the WT plant. Experiments were repeated three times with consistent results. The means of triplicate experiments (*n* = 20) are shown with standard-error bars.

**
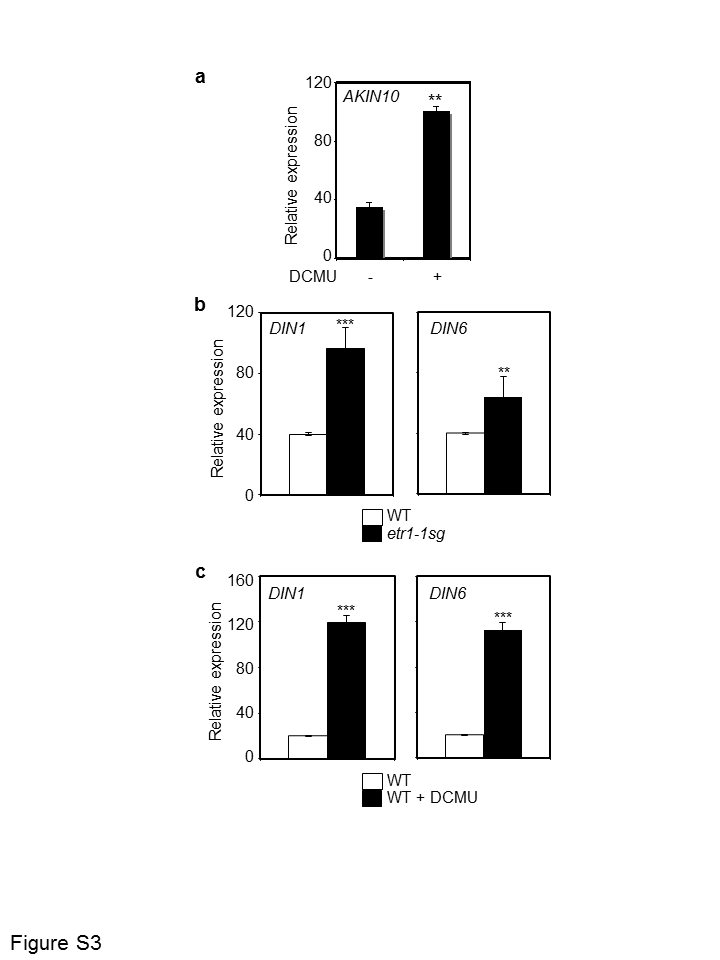
**

**Fig. S4.** *AKIN10* and AKIN10 inducible gene expression in WT in the presence of DCMU or in the mutant with the lack of ethylene responsiveness (**a**) Transcript accumulation of *AKIN10* in the shoots of WT in the presence of DCMU, (**b**,**c**) Transcript accumulation of AKIN10-inducible *DIN1* and *DIN6* in the shoots of WT and *etr1-1* seedlings (**a**), and in WT grown in the presence of DCMU (**b**) Transcript levels were measured using qRT-PCR. All experiments were triplicated with consistent results; the means of triplicate measurements are shown with standard-error bars. ***P < 0.001, **P < 0.01, and *P < 0.05.

**Supplementary table S1. Oligonucleotides used in this study**

Oligo name Oligonucleotide (5′–3′)

**qPCR primers**

*PIF3_f*: CTGAAAGGAGACGGCGTGATAG

*PIF3_r*: CAGATAGTAACCAGACGCCATTGAC

*SUC1_f*: GATACCACAAATGATAGTCTCACTAG

*SUC1_r*: GTTGCCTTGGGTGCGTC

*SUC4_f*: CACCTCATTTGTTGCGTCTC

*SUC4_r*: CTTCTGAATCCTTGTCCGTG

*DIN1_f:* CAGAGTCGGATCAGGAATGG

*DIN1_r:* ATTTGACCGCTCTCACAACC

*DIN6_f:* AACTTGTCGCCAGATCAAGG

*DIN6_r:* GGAACACGTGCCTCTAGTCC

*AKIN10_f1*: ATGAAGTGCAGATGGGTTCC

*AKIN10_r1*: GCAGCACACAGATCCAAGAA

*AKIN10(5’SS)_f1*: CCGAATTTTCTCCTCCGCC

*AKIN10(5’SS)_r1*: CCAATACCAAGATCCAAGAA

*ETR1_f*: ATGTTGACTCATGAGATTAGAAG

*ETR1_r*: CATGCCCTCGTACAGTACC

*UBQ10_f*: AGATCCAGGACAAGGAGGTATTC

*UBQ10_r*: CGCAGGACCAAGTGAAGAGTAG

*TUB4_f*: AGGGAAAGGAAGAGAGGAAG

*TUB4_r*: GCTGGCTAATCCTACCTTTGG

*ELF4a_f*: TCATAGATCTGGTCCTTGAAAC

*ELF4a_r*: GGCAGTCTCTTCGTG CTGAC

Figure. 1 (b)


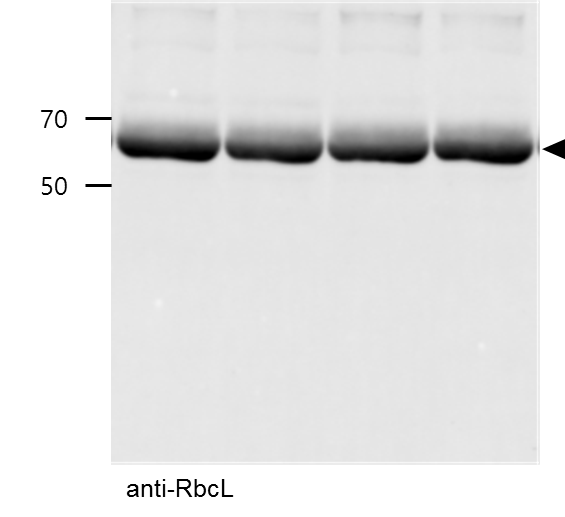


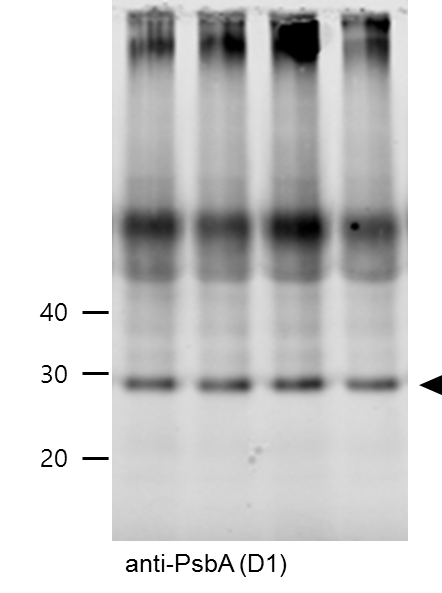


Figure. 5 (a)


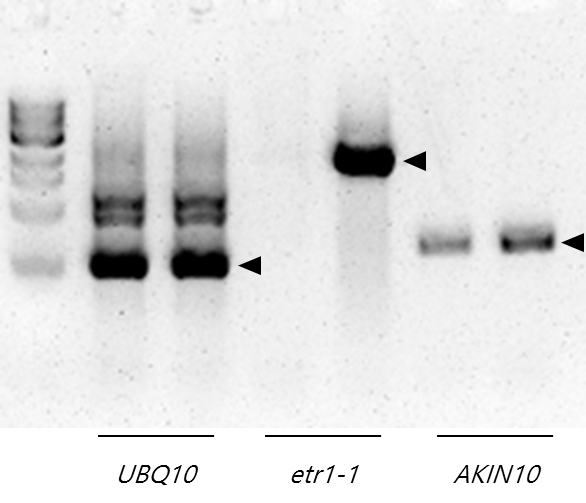

Supplement: Supplementary file 1 — Supplementary information [file 41598_2017_2897_MOESM1_ESM.doc]
